# Metabolic engineering of *Zymomonas mobilis* for co-production of D-lactic acid and ethanol using waste feedstocks of molasses and corncob residue hydrolysate

**DOI:** 10.3389/fbioe.2023.1135484

**Published:** 2023-02-21

**Authors:** Mimi Hu, Weiwei Bao, Qiqun Peng, Wei Hu, Xinyu Yang, Yan Xiang, Xiongying Yan, Mian Li, Ping Xu, Qiaoning He, Shihui Yang

**Affiliations:** ^1^ State Key Laboratory of Biocatalysis and Enzyme Engineering, and School of Life Sciences, Hubei University, Wuhan, China; ^2^ Institute of Modern Physics, Chinese Academy of Sciences, Lanzhou, China; ^3^ Zhejiang Huakang Pharmaceutical Co., Ltd., Kaihua County, China; ^4^ State Key Laboratory of Microbial Metabolism, and School of Life Science and Biotechnology, Shanghai Jiao Tong University, Shanghai, China

**Keywords:** *Zymomonas mobilis*, native CRISPR-Cas system, lactate, beet molasses, corncob residue hydrolysate

## Abstract

Lactate is the precursor for polylactide. In this study, a lactate producer of *Z. mobilis* was constructed by replacing *ZMO0038* with *LmldhA* gene driven by a strong promoter P*adhB*, replacing *ZMO1650* with native *pdc* gene driven by P*tet,* and replacing native *pdc* with another copy of *LmldhA* driven by P*adhB* to divert carbon from ethanol to D-lactate. The resultant strain ZML-pdc-ldh produced 13.8 ± 0.2 g/L lactate and 16.9 ± 0.3 g/L ethanol using 48 g/L glucose. Lactate production of ZML-pdc-ldh was further investigated after fermentation optimization in pH-controlled fermenters. ZML-pdc-ldh produced 24.2 ± 0.6 g/L lactate and 12.9 ± 0.8 g/L ethanol as well as 36.2 ± 1.0 g/L lactate and 40.3 ± 0.3 g/L ethanol, resulting in total carbon conversion rate of 98.3% ± 2.5% and 96.2% ± 0.1% with final product productivity of 1.9 ± 0.0 g/L/h and 2.2 ± 0.0 g/L/h in RMG5 and RMG12, respectively. Moreover, ZML-pdc-ldh produced 32.9 ± 0.1 g/L D-lactate and 27.7 ± 0.2 g/L ethanol as well as 42.8 ± 0.0 g/L D-lactate and 53.1 ± 0.7 g/L ethanol with 97.1% ± 0.0% and 99.1% ± 0.8% carbon conversion rate using 20% molasses or corncob residue hydrolysate, respectively. Our study thus demonstrated that it is effective for lactate production by fermentation condition optimization and metabolic engineering to strengthen heterologous *ldh* expression while reducing the native ethanol production pathway. The capability of recombinant lactate-producer of *Z. mobilis* for efficient waste feedstock conversion makes it a promising biorefinery platform for carbon-neutral biochemical production.

## Introduction

Lactic acid, also known as 2-hydroxypropionic acid, has two isomeric forms: L-lactate and D-lactate (Mazzoli, 2020; Augustiniene et al., 2022). Lactate is usually used as food additives, cosmetics, formulating ointments, anti-acne solutions, humectants, and organic solvents in food, pharmaceutical, chemical and medical industries (Mazzoli, 2020). Recently, the increasing demand of polylactide (PLA) for replacing traditional petroleum-derived plastics drives the global market demand of lactate (Mazzoli, 2020; Augustiniene et al., 2022). PLA has the characteristics of biocompatibility, biodegradability, and elasticity, and can be used for disposable cutlery and trays, packaging, agriculture mulch films, medical products of surgical sutures (Singhvi et al., 2019; Wang, 2021; Augustiniene et al., 2022). About 90% lactate is produced by microbial fermentation with several advantages compared to chemical syntheses, such as lower energy consumption, better environmental protection, and higher purity instead of a racemic mixture of L-lactate and D-lactate (Jung et al., 2010; Zhang et al., 2018).

Current lactate production mainly utilizes edible crops such as corn and sugarcane as feedstocks, which have ethical and economical concerns and threaten the world food supply (Abdel-Rahman et al., 2013; Oliveira et al., 2018). Non-food feedstocks such as molasses and lignocellulosic biomass are promising alternative carbon sources for lactate production (Oliveira et al., 2018; Kong et al., 2019; Palmonari et al., 2020; Lian et al., 2021; Wang, 2021; Svetlitchnyi et al., 2022). Molasses is a world-widely used by-product from sugarcane and beet extractions (Palmonari et al., 2020). Lignocellulosic biomass is the largest and commonly used fraction of waste biomass, including corncob residues, corn stover, sugar-cane bagasse, and wood processing waste (Oliveira et al., 2018; Mazzoli, 2020; Mazzoli, 2021). For example, about 23 million metric tons corncob residues are available annually for alternative uses in China alone (Sun et al., 2011; Liu et al., 2016).

Lactate is mainly produced by lactic acid bacteria (LAB) like *Lactobacillus* and *Lactococcus* as well as potential natural lactate producers belonging to the genus of *Bacillus* and *Rhizopus* (Augustiniene et al., 2022). However, these strains cannot utilize lignocellulosic biomass efficiently (Abedi and Hashemi, 2020; Augustiniene et al., 2022). Although various metabolic engineering strategies have been applied to produce lactate efficiently from lignocellulosic biomass, such as enhancing the expression or activity of enzymes of lactate biosynthesis pathway, disrupting pathways that compete for carbon substrate, electrons and co-factors, as well as enhancing strain acid-stress tolerance by overexpressing the transporter related genes (Li et al., 2017; Weusthuis et al., 2017; Zhang et al., 2017; Kong et al., 2019; Tsuge et al., 2019; Zhu et al., 2019), the large-scale industrial production of lactate from cost-effective feedstocks has not yet been commercialized.

*Zymomonas mobilis* is a natural ethanologenic bacterium with desirable industrial characteristics such as generally regarded as safe (GRAS), high sugar uptake efficiency and conversion rate, low by-product, phage free and no need to control aeration during fermentation (Yang Q. et al., 2020a; Yang et al., 2021). Various native and heterologous metabolic pathways have been enhanced or constructed using the established CRISPR-Cas genome editing toolkits (Shen et al., 2019; Zheng et al., 2019; Yang Y. et al., 2020b) for desirable bioproducts, such as isobutanol, 2,3-butanediol, polyhydroxybutyrate (PHB) and lactate (Yang et al., 2016; Liu et al., 2020; Yang et al., 2021; Li et al., 2022). In addition to pure sugars, lignocellulosic hydrolysates can also be utilized as carbon sources by *Z. mobilis* (Yang et al., 2016; Wang et al., 2018; Todhanakasem et al., 2019).

*Z. mobilis* ZM4 has two homologous proteins encoding lactate dehydrogenase (ZMO0256 and ZMO1237) and can produce less than 1 g/L lactate (Yang et al., 2016; Martien et al., 2019). Lactate production can be further increased when heterologous lactate dehydrogenase genes were introduced into the host. For example, the recombinant *Z. mobilis* CP4 and *Z. mobilis* ZM4 produced 10.8 g/L and 2.1 g/L L-lactate by introducing heterologous genes encoding lactate dehydrogenase from *Lactobacillus casei* and *Bacillus coagulans*, respectively (Jiang et al., 2011; Shen et al., 2019). Since ethanol is the main by-product in lactate production, knocking out of pyruvate decarboxylase gene (*pdc*) becomes an efficient way to block the ethanol production pathway. However, it is difficult to knock out the essential chromosomal gene *pdc* unless another *pdc* copy was provided. For example, a lactate-producing recombinant strain of *Z. mobilis* was constructed by introducing heterologous D-lactate dehydrogenase *ldhA* gene from *E. coli,* deleting the native *pdc* gene, and introducing a copy of *pdc* gene under the control of IPTG-inducible promoter. The resultant recombinant strain can produce 14 g/L lactate (Liu et al., 2020).

In this study, a stable lactate producer of *Z. mobilis* ZM4 recombinant strain was constructed by introducing a heterologous *LmldhA* gene from *Leuconostoc mesenteroides* into the chromosome of *Z. mobilis* while reducing the expression of *pdc* to divert carbon from ethanol biosynthesis to lactate production using the native CRISPR-Cas genome editing toolkit. Subsequently, lactate fermentation conditions were optimized, and D-lactate production of recombinant strain was investigated using low-cost waste feedstocks of beet molasses and corncob residue hydrolysate.

## Materials and methods

### Strains, media, and growth conditions

The wild-type *Z. mobilis* ZM4 (ATCC 31821) strain and its derivative strains were cultured in Rich Medium (RM: 10 g/L yeast extract, 1 g/L KH_2_PO_4_, 1 g/L K_2_HPO_4_) with 50 g/L glucose (RMG5, pH 5.80) or 120 g/L glucose (RMG12, pH 5.80) at 30 °C, 100 rpm. *Escherichia coli* DH5α, used for plasmid construction, was grown in Luria-Bertani medium (LB: 10 g/L NaCl, 10 g/L tryptone, 5 g/L yeast extract, and 1.5% agar for solid) at 37°C, 250 rpm. The antibiotics of spectinomycin (100 μg/mL) and chloramphenicol (100 μg/mL) were used for *E. coli* or *Z. mobilis* when required, respectively.

The 20% beet molasses (BM) used in this study contains 77.1 g/L sucrose, 4.8 g/L glucose, 6.8 g/L fructose, and 6.3 g/L lactate, pH 5.93. The original corncob residue hydrolysate (CRH) was provided by ZheJiang HuaKang Pharmaceutical Co., Ltd. (Zhejiang, China), which contains 150 g/L glucose, 19.5 g/L xylose, and 2.1 g/L acetic acid, pH 4.35. The CRH used in present study was supplemented with 10 g/L yeast extract, 1 g/L KH_2_PO_4_ and 1 g/L K_2_HPO_4_.

### Construction of plasmids and recombinant strains

A heterologous gene *LmldhA* (AB233384.1) encoding D-lactate dehydrogenase derived from *L. mesenteroides* was synthesized from GenScript (Nanjing, China). And the 370-bp sequence in front of the alcohol dehydrogenase gene (*ZMO1596*) was chose as the constitutive promoter P*adhB*. Then, *LmldhA* and P*adhB* were amplified with the primers of *LmldhA*-F/R and P*adhB*-F/R, respectively. The *LmldhA* driven by P*adhB* was constructed as a 1366-bp *LmldhA*-expressing cassette through overlapping extension PCR and further cloned into the shuttle vector pEZ15Asp (Yang et al., 2016) to generate plasmid pEZ-ldh. Lastly, the plasmid pEZ-ldh was electroporated into ZM4 using a Bio-Rad Gene Pulser (Bio-Rad, CA, United States). Electroporated cells were recovered in RMG5 for 3 h and then spread on RMG5 agar plates containing 100 μg/mL spectinomycin for 2-days incubation at 30°C. Transformants were selected by colony PCR with primers of pEZ15A-F/R, and confirmed by Sanger sequencing at Sangon Biotech (Shanghai, China) to obtain recombinant strain ZM4 (pEZ-ldh).

### Construction of editing plasmids and mutants

The chromosomal locus of *ZMO0038* and *ZMO1650* were selected as gene integration location since their deletions do not affect the growth of *Z. mobilis* (Yang et al., 2016; Qiu et al., 2020). Here, the integration of *ZMO0038* with *LmldhA* was presented as an example. Briefly, the 32-bp spacer sequences (gRNA-*0038*-F and gRNA-*0038*-R) were designed and ordered from TsingKe Biotechnology Co., Ltd. (Beijing, China). The oligonucleotides of spacers were annealed and ligated into the linearized pL2R plasmid (Zheng et al., 2019) with *Bsa* I digestion, and the resulting plasmid was named pL2R-g*0038*. Subsequently, donor DNA fragments, each containing 800-bp upstream and downstream sequences of *ZMO0038* were amplified with the primers of up-*0038*-F/R and down-*0038*-F/R. And pL2R-g*0038* was amplified with pL2R-FK-F/R for linearization. Then, the donor DNA fragments and *LmLdhA-*expressing cassette were cloned into linearized pL2R-g*0038* by T5 exonuclease (NEB, WA, United States) (Zheng et al., 2019). The resultant *ZMO0038*-replacing editing plasmid was PCR confirmed with pEZ15A-F/R, and the correct plasmid was named as pRep-*0038* (*ldh*). Similarly, editing plasmids of pRep-*1650* (*ldh*), pRep-*1650* (*pdc*) and pRep-*1360* (*ldh*) were constructed for replacement of *ZMO1650* with *LmldhA*, replacement of *ZMO1650* with *ZMO1360* (*pdc*), and replacement of *ZMO1360* (*pdc*) with *LmldhA*, respectively.

The editing plasmid pRep-*0038* (*ldh*) was then electroporated into *Z. mobilis* ZM4. Electroporated cells were then spread on RMG5 agar plates containing 100 μg/mL spectinomycin and incubated at 30 °C for 2–3 days. Single colonies were selected based on colony PCR results using the primers of Chk-*0038*-F/R, and confirmed by Sanger sequencing at Sangon Biotech (Shanghai, China).

### Curing of editing plasmids

Transformants with correct PCR results were cultivated in RMG5 plates and screened using primers pEZ15A-F/R to cure the editing plasmid. The loss of the editing plasmids for recombinant strains ZML, ZML-ldh, ZML-pdc, and ZML-pdc-ldh were further confirmed by colony PCR. All primers used in this work were provided in Supplementary Table S1.

### Shake flask and batch fermentation

Strains were cultured in a 50 mL flask containing 40 mL RMG5 at 30 °C in an orbital incubator shaker at a speed of 100 rpm with an initial OD_600 nm_ value of 0.1. Batch fermentation was conducted in 1-L bioreactor (T&J Bio-engineering Co., Ltd., Shanghai, China) with 0.6 L RMG5, RMG12, BM, or CRH. The temperature and agitation were set at 30 °C and 100 rpm, respectively. pH was set at a constant value of 5.8 using 4 M KOH during batch fermentation for RM and BM. As for batch fermentation using CRH, 10 g/L CaCO_3_ was supplemented in the medium at the beginning for pH adjustment. The initial OD_600 nm_ of 0.1 was used for fermentation using RM, and initial OD_600 nm_ of 0.5 for fermentation using BM and CRH, respectively.

### Fermentation analysis

During fermentation, the cultures were sampled at different time points of post-inoculation to monitor cell growth and concentrations of sucrose, glucose, fructose, ethanol, and lactate. Cell growth in terms of its optical density at 600 nm was monitored with a UV–visible spectrophotometer UV-1800 (AoYi Instrument Co., Ltd., Shanghai, China). Samples from the shake flasks or bioreactors were centrifuged at 13,000 rpm for 2 min and then the supernatants were filtered through a 0.2-μm syringe filter into high-performance liquid chromatography (HPLC) vials. Concentrations of sucrose, glucose, fructose, ethanol, and lactate in the supernatants were then detected by HPLC (Shimadzu, Japan) equipped with a refractive index detector (RID) and a column (300 × 7.8 mm) of Bio-Rad Aminex HPX-87H (Hercules, CA, USA) with 5 mM H_2_SO_4_ as the mobile phase at a flow rate of 0.5 mL/min, column temperature of 65 °C, and an injection volume at 20 μL as previously described (Hu et al., 2021).

### Calculation of carbon conversion efficiency

The total consumed sugar (*C*_*Total*_) was calculated as follows (Hu et al., 2021):

$$C_{Total}=\left(S\;*\;0.526+G\right)+\left(S\;*\;0.526+F\right)$$

In this equation, “S” means sucrose consumed, “G” means glucose consumed, and “F” means fructose consumed. “0.526” is the theoretical yield from sucrose into glucose and fructose.

The sugar consumed for ethanol and lactate production are calculated according to the following formulas, respectively:

Cethanol=g ethanol/0.511

CLA=g LA/1

“0.511” and “1” represent the theoretical yields of ethanol and lactate from glucose, respectively (Liu et al., 2020).

The carbon conversion efficiency (η_c_, %) represents the efficiency to convert all sugars into lactate and ethanol, which can be calculated as follows (Lian et al., 2021):

$$\eta_{c}=\left(C_{ethanol}+C_{LA}\right)/\;C_{Total}$$

### Statistical analysis

Data presented in the graphs were performed with the mean ± SD and T-tests value using the GraphPad Prism statistical software (version 8.0.1). P < 0.05 was considered with statistically significant difference.

## Results

### Construction of heterologous lactate-producing strains of *Z. mobilis*

**FIGURE 1:**
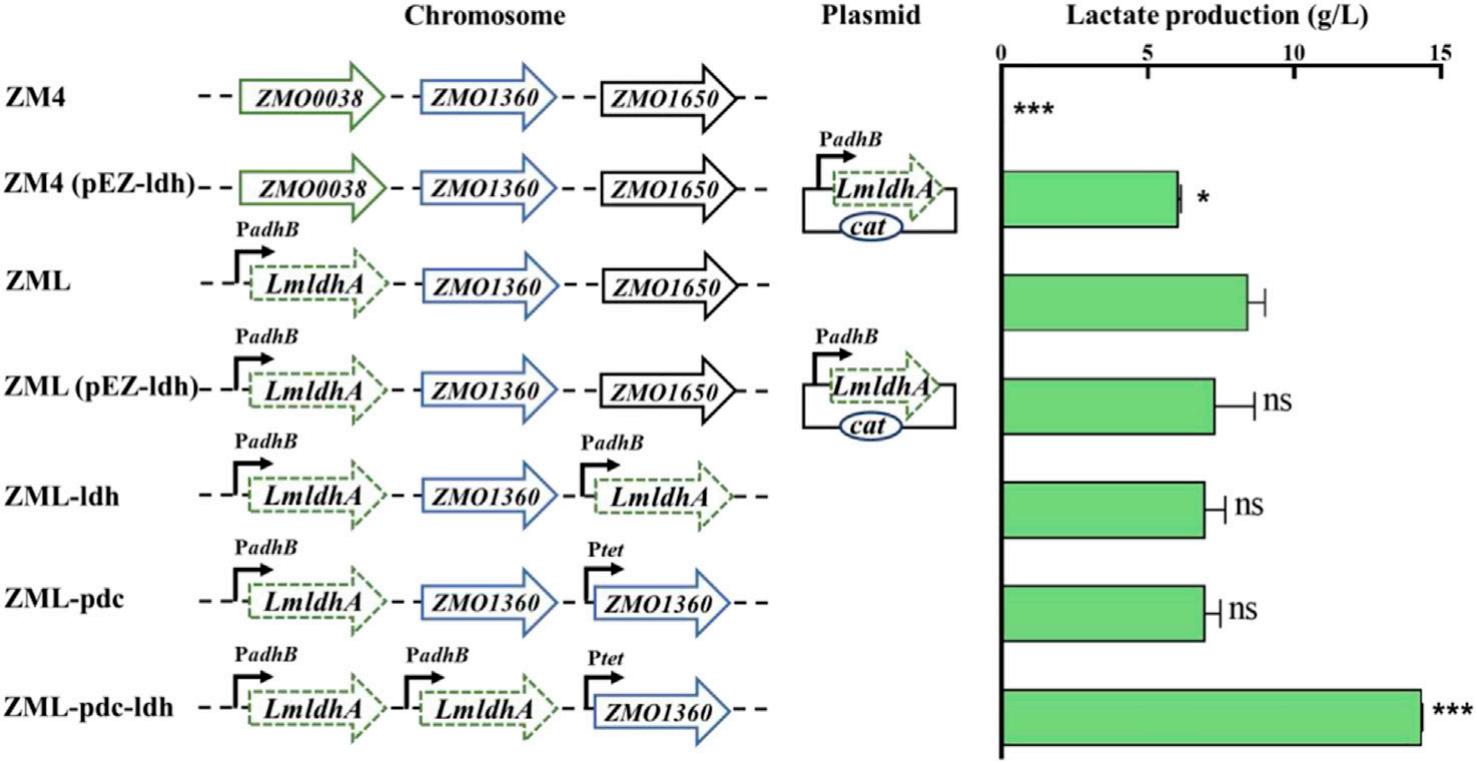
Construction and evaluation of lactate-production recombinant strains of *Z. mobilis*. T-test analysis was conducted for ZML with ZM4, ZM4 (pEZ-ldh), ZML (pEZ-ldh), ZML-ldh, ZML-pdc, and ZML-pdc-ldh. ns represents no significant difference (*p*-value > 0.05), * represents a significant difference with *p*-value < 0.05. *** represents a significant difference with *p*-value < 0.001. Three replicates were performed for the experiment.

*LmldhA* gene from *L. mesenteroides* subsp*. mesenteroides* ATCC 8293 was selected for constructing lactate-producing strain of *Z. mobilis* in this study, which had a high specific activity when expressed in *Saccharomyces cerevisiae* (Li et al., 2012; Baek et al., 2016). *LmldhA* gene driven by the native strong promoter P*adhB* of *Z. mobilis* was cloned into the shuttle vector pEZ15Asp to obtain the plasmid pEZ-ldh, which was then transformed into ZM4 to generate ZM4 (pEZ-ldh) (Figure 1). ZM4 (pEZ-ldh) produced 6.0 ± 0.0 g/L D-lactate in medium containing 50 g/L glucose, which was significantly higher than that of 0.5 ± 0.1 g/L lactate in the parental strain ZM4 (Table 1). However, the growth rate of 0.29 ± 0.00 h^-1^ in ZM4 (pEZ-ldh) was lower than that of 0.39 ± 0.01 h^-1^ in ZM4. The lactate accumulation in recombinant strain ZM4 (pEZ-ldh) may confront with several negative effects, such as the oxidative stress from the accumulated lactate and the acidic medium (Peetermans et al., 2021).

**TABLE 1:** Fermentation performance of the growth rate, glucose consumption, the yields of D-lactate and ethanol, as well as the carbon conversion rate of wild-type *Z. mobilis* ZM4 and its derivative strains of ZM4 (pEZ-ldh), ZML, ZML (pEZ-ldh), ZML-ldh, ZML-pdc, and ZML-pdc-ldh in RMG5.

Strain	Time (h)	Growth rate (h^-1^)	Glucose consumption (g/L)	D-lactate (g/L)	Ethanol (g/L)	Carbon conversion rate (%)
**ZM4**	11	0.39 ± 0.01	49.8 ± 0.0	0.5 ± 0.1	25.1 ± 0.1	98.9 ± 0.7
**ZM4 (pEZ-ldh)**	36	0.29 ± 0.00	49.8 ± 0.0	6.0 ± 0.0	20.3 ± 0.2	93.8 ± 2.4
**ZML**	25	0.26 ± 0.02	49.8 ± 0.0	8.1 ± 0.0	21.0 ± 0.0	98.8 ± 0.3
**ZML (pEZ-ldh)**	25	0.22 ± 0.00	49.7 ± 0.2	7.3 ± 1.3	21.2 ± 1.0	98.3 ± 1.5
**ZML-ldh**	25	0.27 ± 0.01	49.9 ± 0.0	7.0 ± 0.7	21.0 ± 1.5	96.5 ± 4.6
**ZML-pdc**	25	0.23 ± 0.00	49.6 ± 0.1	6.9 ± 0.5	21.5 ± 1.4	95.6 ± 4.6
**ZML-pdc-ldh**	44	0.18 ± 0.00	48.0 ± 0.9	13.8 ± 0.2	16.9 ± 0.3	98.3 ± 1.7

To construct a stable lactate-producing strain, *ZMO0038* of *Z. mobilis* ZM4 was replaced by P*adhB*-*LmldhA*, a construct containing *LmldhA* gene driven by P*adhB* promoter, using the native type I-F CRISPR-Cas genome editing system to generate a lactate-producing recombinant strain ZML (Figure 1). Our result exhibited that ZML can produce 8.1 ± 0.0 g/L D-lactate in medium containing 50 g/L glucose, which was 1.35 times higher than that of ZM4 (pEZ-ldh) with 6.0 ± 0.0 g/L D-lactate (Table 1). It took ZML 25 h to consume all glucose compared to that of 36 h for ZM4 (pEZ-ldh) (Table 1), which indicated that ZML finished lactate fermentation faster than ZM4 (pEZ-ldh).

To further compete with ethanol production for high lactate production, two recombinant strains were constructed by increasing the copy number of *LmldhA* gene. ZML (pEZ-ldh) was constructed by introducing the pEZ-ldh plasmid into ZML, and ZML-ldh was constructed by integrating another copy of *LmldhA* gene to replace the chromosomal gene *ZMO1650* (Figure 1). However, ZML (pEZ-ldh) and ZML-ldh produced 7.3 ± 1.3 g/L and 7.0 ± 0.7 g/L D-lactate, which were lower than that of ZML with a titer of 8.1 ± 0.0 g/L D-lactate (Table 1). More importantly, the ethanol production was unaffected. Thus, the carbon flux cannot be diverted to produce lactate by increasing the copy number of heterologous D-lactate dehydrogenase *LmldhA* gene alone in this study.

We then attempted to redirect carbon flux from ethanol to lactate by replacing the pyruvate decarboxylase gene *ZMO1360* (*pdc*) driven by its strong promoter with an inducible promoter P*tet* from the vector Ptet_Dual_Spe (MW812440) (Yang et al., 2019). *Pdc* gene driven by P*tet* was first integrated into the locus of chromosomal gene *ZMO1650* to generate the resultant strain ZML-pdc (Figure 1), which produced 6.9 ± 0.5 g/L D-lactate compared to 8.1 ± 0.0 g/L in ZML. Subsequently, *pdc* gene driven by its native strong promoter was replaced by *LmldhA* under the control of P*adhB* promoter to generate the recombinant strain ZML-pdc-ldh (Figure 1). ZML-pdc-ldh produced 13.8 ± 0.2 g/L D-lactate after all 50 g/L glucose was consumed 44 h post-inoculation (Table 1). In addition, less ethanol was produced in ZML-pdc-ldh (16.9 ± 0.3 g/L) compared with ZML (21.0 ± 0.0) and *Z. mobilis* ZM4 (25.1 ± 0.1 g/L) in flasks, respectively (Table 1). It thus demonstrated that the strategy of diverting the carbon into ethanol was effective with about 20–30% ethanol production reduced for lactate production.

A recombinant strain *Zmo*-LdhA was reported in a previous study, which can produce 14 g/L lactate in pH-controlled bioreactor when Pdc was repressed in the absence of IPTG and LdhA was induced with 400 nM tetracycline (Liu et al., 2020). In current study, no extra inducer was utilized, and ZML-pdc-ldh can produce 13.8 ± 0.2 g/L D-lactate in flasks without pH control during fermentation. Thus, ZML-pdc-ldh was selected for further experiments.

### Optimization of D-lactate fermentation conditions of *Z. mobilis*

Compared with the growth rate of 0.29 h^-1^ for ZM4 (pEZ-ldh), the growth rate of ZML-pdc-ldh was only 0.18 h^-1^, which might be attributed to the acidic environment resulting from lactate accumulation that have been reported in other microorganisms such as *Lactobacillus lactis* and *S. cerevisiae* (Yang et al., 2015; Peetermans et al., 2021). The acidic pH environment has an impact on cell functions including DNA and RNA synthesis and many metabolic processes (Warnecke and Gill, 2005; Yang Q. et al., 2020a; Peetermans et al., 2021), and the addition of neutralizer like CaCO_3_ to balance the pH is usually applied in lactate fermentation (Yen et al., 2010).

**FIGURE 2:**
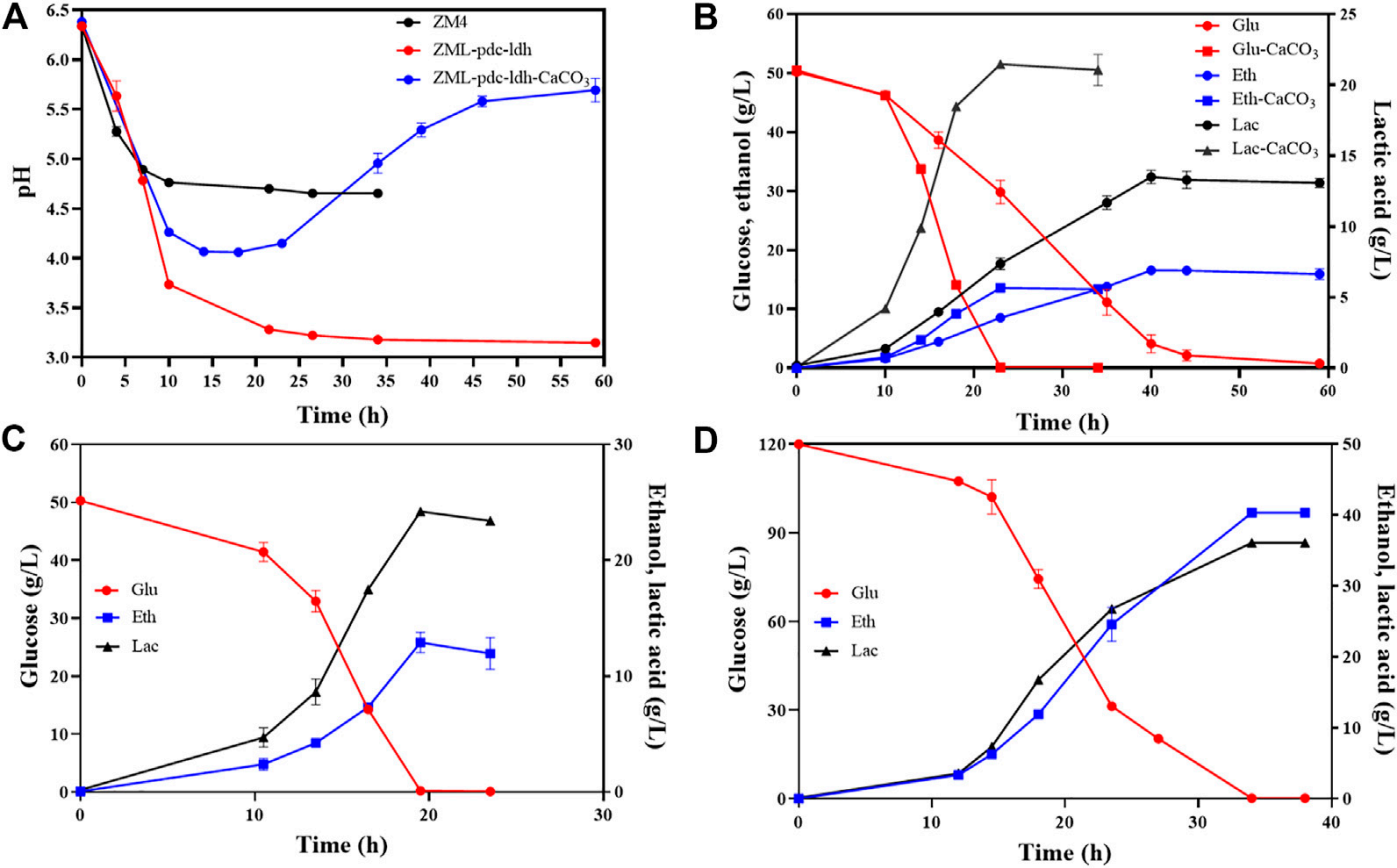
pH changes of wild-type ZM4 and the recombinant strain ZML-pdc-ldh during fermentation **(A)**, and glucose consumption, lactate and ethanol production of ZML-pdc-ldh **(B)** in the presence or absence of CaCO_3_, as well as glucose consumption, lactate and ethanol production of ZML-pdc-ldh in RMG5 **(C)** or RMG12 **(D)** in pH-controlled fermenters. Glu, Eth and Lac represent glucose, ethanol, and lactate, respectively. Three replicates were performed for the experiment.

Therefore, the influence of CaCO_3_ addition on D-lactate production in strain ZML-pdc-ldh was investigated. First, 10 g/L CaCO_3_ was supplemented in the medium at the beginning of the fermentation, and the changes of pH values in the medium of wild-type strain ZM4 and ZML-pdc-ldh were monitored. The pH values of the media in both strains dropped without the addition CaCO_3_. And the pH was kept at 4.6 for ZM4, while it sharply declined to pH value lower than 4.0 after 10 h post-inoculation, and continuous to drop to nearly 3.0 at the end of the fermentation for ZML-pdc-ldh (Figure 2A).

With the addition of CaCO_3_, the pH of ZML-pdc-ldh medium decreased at first 14 h and then increased from 4.0 to 5.7, which was consistent with the result observed in yeast *Candida sonorensis* (Ilmén et al., 2013). In addition, the glucose consumption, lactate, and ethanol production were determined during the fermentation process with CaCO_3_ addition. When glucose was completely consumed 22 h post-inoculation, 21.5 ± 0.2 g/L D-lactate and 13.6 ± 0.1 g/L ethanol produced (Figure 2B). Compared with the result of 13.8 ± 0.2 g/L D-lactate and 16.9 ± 0.3 g/L ethanol produced in RMG5 without CaCO_3_ for 44 h cultivation, it demonstrated that more carbon can be diverted into D-lactate in ZML-pdc-ldh with the addition of CaCO_3_ as neutralizing agent to balance pH in culture medium (Figure 2B).

Lactate fermentation in pH-controlled fermenters was further tested for ZML-pdc-ldh. Batch fermentation with different glucose concentration (RMG5: 50 g/L and RMG12: 120 g/L) in 1-L bioreactor at a constant pH of 5.8 was conducted. After glucose was completely consumed within 19 h for RMG5 (Figure 2C) and 34 h for RMG12 (Figure 2D), 24.2 ± 0.6 g/L and 36.2 ± 1.0 g/L D-lactate accumulated, and 12.9 ± 0.8 g/L and 40.3 ± 0.3 g/L ethanol were generated, resulting in a total carbon conversion rate of 98.3% ± 2.5% and 96.2% ± 0.1% with the final product productivity of 1.9 ± 0.0 g/L/h and 2.2 ± 0.0 g/L/h in RMG5 and RMG12, respectively (Table 2).

**TABLE 2:** Batch fermentation performance of total sugar consumption time, lactate and ethanol titers, total carbon conversion rate, and final product productivity of ZML-pdc-ldh in RMG5, RMG12, 20% BM (Beet molasses), and CRH (Corncob residue hydrolysate).

Medium	Time (h)	Total sugar consumption (g/L)	D-lactate (g/L)	Ethanol (g/L)	Carbon conversion rate (%)<	Final product productivity (g/L/h)
**RMG5**	19	49.8 ± 0.0	24.2 ± 0.6	12.9 ± 0.8	98.3 ± 2.5	1.9 ± 0.0
**RMG12**	34	119.8 ± 0.1	36.2 ± 1.0	40.3 ± 0.3	96.2 ± 0.1	2.2 ± 0.0
**20% BM**	110	89.7 ± 0.5	32.9 ± 0.1	27.7 ± 0.2	97.1 ± 0.0	0.6 ± 0.0
**CRH**	45	148.1 ± 0.1	42.8 ± 0.0	53.1 ± 0.7	99.1 ± 0.8	2.1 ± 0.0

### D-lactate production using molasses and waste corncob residue hydrolysate

**FIGURE 3:**
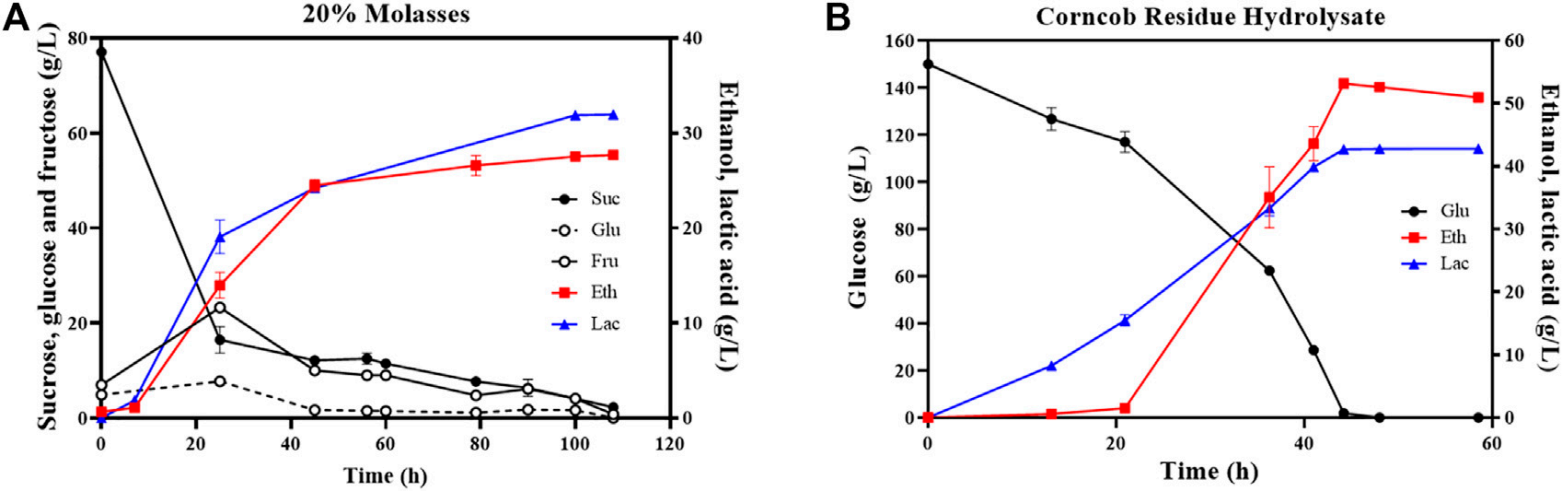
Batch fermentation of ZML-pdc-ldh in 20% beet molasses **(A)** and corncob residue hydrolysate **(B)**. Glu, Eth, and Lac represent glucose, ethanol, and lactate, respectively. Three replicates were performed for the experiment.

20% beet molasses (BM) without yeast extract supplementation and sterilization process was conducted anaerobically using ZML-pdc-ldh in a 1-L bioreactor at pH 5.8. As demonstrated in Figure 3A, the concentration of total sugar including sucrose, glucose, and fructose was dramatically decreased at first 45 h from 92.9 ± 0.3 g/L to 24.5 ± 0.8 g/L. After 110 h fermentation, 32.9 ± 0.1 g/L D-lactate and 27.7 ± 0.2 g/L ethanol were produced by ZML-pdc-ldh (Figure 3A). Although the productivity of final product of 0.6 ± 0.0 g/L/h in 20% BM was significantly decreased due to the long time for sugars utilization, a high carbon conversion rate of 97.1% ± 0.0% was achieved (Table 2).

However, it is notable that the time for sugar consumption of ZML-pdc-ldh in 20% molasses significantly increased for more than 5.8 folds or 3.2 folds when compared with the conditions under RMG5 (19 h) or RMG12 (34 h), respectively. It might be ascribed to that sucrose is the main sugar composition of beet molasses, accounting for 81.16%. Sucrose is a disaccharide that needs to be hydrolyzed into monosaccharides of glucose and fructose for utilization during fermentation. More importantly, uptakes of these two sugars are usually competed, and both can be consumed as major substrate and converted to glucose 6-phosphate for *Z. mobilis* with different fermentation pathways (Palmonari et al., 2020; Braga et al., 2021). Therefore, considering the time for sucrose hydrolysis as well as transportation and catabolism of different sugars, longer time is needed than that of using monosaccharides. Additionally, mixed sugar monomers and oligomers with low quantities, organic acids (i.e., lactic, acetic, butyric, propionic, citric, and malic acids), and other components (i.e., sulfates, phosphates, chlorides, and nitrates) existing in beet molasses may also have negative effects on efficient sugar consumption (Dumbrepatil et al., 2008; Palmonari et al., 2020).

As for corncob residue hydrolysate (CRH) containing 150 g/L glucose, 19.5 g/L xylose, and 2.1 g/L acetic acid, glucose was completely utilized by ZML-pdc-ldh in a 1-L bioreactor after 45 h post-inoculation to produce 42.8 ± 0.0 g/L D-lactate and 53.1 ± 0.7 g/L ethanol (Figure 3B). The total carbon conversion rate of 99.1% ± 0.8% in CRH was the highest among all tested substrates in this study. And the value of 2.1 g/L/h final product productivity in CRH was comparable with the results under glucose fermentation, which was 1.9 g/L/h or 2.2 g/L/h in RMG5 or RMG12, respectively (Table 2).

Moreover, the batch fermentation using CRH by ZML-pdc-ldh was simple and convenient without sterilization and aeration. It also did not need to supplement nutrients such as amino acids, nucleotides and/or vitamins that are usually needed by several lactic acid bacteria for fermentation (Kylä-Nikkilä et al., 2000; Abedi and Hashemi, 2020; Ma et al., 2022). All these results demonstrated that *Z. mobilis* can utilize corncob residue hydrolysates as suitable carbon resource for economic lactate production for sustainable bioeconomy and environment protection.

## Conclusion

In this study, a stable heterologous D-lactate producing strain of ZML-pdc-ldh was constructed and optimized for lactate production. The results demonstrated that recombinant ZML-pdc-ldh can divert 20%–30% carbon from ethanol production to produce 13.8 ± 0.2 g/L D-lactate in flask fermentations. ZML-pdc-ldh produced 24.2 ± 0.6 g/L lactate and 12.9 ± 0.8 g/L ethanol as well as 36.2 ± 1.0 g/L lactate and 40.3 ± 0.3 g/L ethanol, resulting in total carbon conversion rate of 98.3% ± 2.5% and 96.2% ± 0.1% with the final product productivity of 1.9 ± 0.0 g/L/h and 2.2 ± 0.0 g/L/h in RMG5 and RMG12, respectively. More importantly, ZML-pdc-ldh had high total carbon conversion rates all above 97% using waste feedstocks of molasses and corncob residue hydrolysates to produce lactate and ethanol efficiently. The production of 32.9 ± 0.1 g/L D-lactate and 27.7 ± 0.2 g/L ethanol was obtained using 20% molasses, and 42.8 ± 0.0 g/L D-lactate and 53.1 ± 0.7 g/L ethanol was obtained using corncob residue hydrolysate with the final product productivity of 0.6 ± 0.0 g/L/h and 2.1 ± 0.0 g/L/h, respectively. More importantly, ZML-pdc-ldh had high total carbon conversion rates all above 97% in both waste feedstocks without the needs of sterilization, aeration, and the supplementation of expensive nutrients and extra inducer. This work thus provides a strategy for harnessing waste feedstocks for co-production of carbon-neutral D-lactate and ethanol in *Z. mobilis*. Future studies can be carried out to develop ZML-pdc-ldh as a sole lactate producer by channeling carbon from ethanol production into lactate production completely.

## Data Availability

The original contributions presented in the study are included in the article/Supplementary Material; further inquiries can be directed to the corresponding authors.
