# Supplementary material for: Metabolic engineering of Zymomonas mobilis for co-production of D-lactic acid and ethanol using waste feedstocks of molasses and corncob residue hydrolysate
Source: Front Bioeng Biotechnol. 2023 Feb 21;11:1135484. doi: 10.3389/fbioe.2023.1135484 (PMC9989019; doi:10.3389/fbioe.2023.1135484)
Supplement: Supplementary file 1 [file Table1.DOCX]

**Table S1. List of primers used in the study.**

| **Primer** | **Sequence (5’-3’)** |
| --- | --- |
| *LmldhA*-F | ggtgaggttatagctatgaagatttttgcttacggcattc |
| *LmldhA*-R | taatattcaacagcaatagctggcttctcac |
| P*adhB*-F | ttcataatttgcataagtcttgatgtaaaaaatac |
| P*adhB*-R | agctataacctcaccctacatactag |
| pEZ15A-F | ggcaaagccaccctatttttag |
| pEZ15A-R | cacttcactgacaccctcat |
| gRNA-*0038*-F | gaaacaaatgcctaagcgcctctgtcactttcggta |
| gRNA-*0038*-R | gaactaccgaaagtgacagaggcgcttaggcatttg |
| up-*0038*-F | tcaccagctcaccgtctgtatcgcgccccaatatgaccg |
| up-*0038*-R | cttgactccctccatgcacttaaaaaatc |
| down-*0038*-F | tcgttaaatattcagatagacggagataataaacggga |
| down-*0038*-R | gagagatctgatatcactttaggcgagaagggaaagggca |
| pL2R-FK-F | agtgatatcagatctcgagctcggtacccgg |
| pL2R-FK-R | agacggtgagctggtgacct |
| Chk-*0038*-F | aggatggtcgatcttcagctattgtg |
| Chk-*0038*-R | gtgaaccgccaaaaactcgg |
| gRNA-*1650*-F | gaaattcaaaagaagtattggtaagcgagaccacgg |
| gRNA-*1650*-R | gaacccgtggtctcgcttaccaatacttcttttgaa |
| up-*1650*-F | gtcaccagctcaccgtctccgatccgccctatggtct |
| up-*1650*-R | ggaggatattccagagaagaaagtaagcaatc |
| down-*1650*-F | ggtgcggtcttgattagccttgaa |
| down-*1650*-R | tcgagatctgatatcactgtgctatccgcttggctctc |
| P*tet*-F | acggtctcccgtttaagaccc |
| P*tet*-R | gggagatcctttctcctctttagatcatttgaatacttttct |
| *pdc*-F | gaggagaaaggatctcccatgagttatactgtcggtacctatttagcg |
| *pdc*-R | gctaatcaagaccgcaccctagaggagcttgttaacaggcttacg |
| Chk-*1650*-F | ccgatggcaaaatctgggttg |
| Chk-*1650*-R | taccgagacgggaaagacag |
| gRNA-*1360*-F | gaaatatagctaaatccggaacgacactttagaggt |
| gRNA-*1360*-R | gaacacctctaaagtgtcgttccggatttagctata |
| up-*1360*-F | gtcaccagctcaccgtctattgtaggcggctggattgt |
| up-*1360*-R | ggaatcagaaccagatgtagaaacgtagc |
| down-*1360*-F | tttttaaataaacttagagcttaaggcgaa |
| down-*1360*-R | tcgagatctgatatcacttacctgattacgacaaatcaagcag |
| Chk-*1360*-F | tgctgacaaaaggggacatga |
| Chk*-1360*-R | acttgaataaaccgccacaga |
